# Development of a Clinical Decision Rule for the Early Safe Discharge of Patients with Mild Traumatic Brain Injury and Findings on Computed Tomography Brain Scan: A Retrospective Cohort Study

**DOI:** 10.1089/neu.2019.6652

**Published:** 2019-12-20

**Authors:** Carl Marincowitz, Fiona E. Lecky, Victoria Allgar, Peter Hutchinson, Hadir Elbeltagi, Faye Johnson, Eimhear Quinn, Silvia Tarantino, Will Townend, Angelos G. Kolias, Trevor A. Sheldon

**Affiliations:** ^1^ Hull York Medical School, University of Hull, Hull, United Kingdom.; ^2^ School of Health and Related Research, University of Sheffield, Sheffield, United Kingdom.; ^3^ Hull York Medical School, John Hughlings Jackson Building, University of York, Heslington, United Kingdom.; ^4^ Division of Neurosurgery, Department of Clinical Neurosciences, Addenbrooke's Hospital and University of Cambridge, Cambridge, United Kingdom; NIHR Global Health Research Group on Neurotrauma, University of Cambridge, Cambridge, United Kingdom.; ^5^ Emergency Department, Salford Royal Hospital, Salford Royal NHS Foundation Trust, Salford, United Kingdom.; ^6^ Salford Royal Hospital, Acute Research Delivery Team, Salford Royal NHS Foundation Trust, Salford, United Kingdom.; ^7^ Emergency Department, Hull University Teaching Hospitals NHS Trust, Hull, United Kingdom.; ^8^ Department of Health Sciences, University of York, Alcuin Research Resource Centre, Heslington, United Kingdom.

**Keywords:** intracranial hemorrhage, mild traumatic brain injury, minor head injury, prognostic modeling

## Abstract

International guidelines recommend routine hospital admission for all patients with mild traumatic brain injury (TBI) who have injuries on computed tomography (CT) brain scan. Only a small proportion of these patients require neurosurgical or critical care intervention. We aimed to develop an accurate clinical decision rule to identify low-risk patients safe for discharge from the emergency department (ED) and facilitate earlier referral of those requiring intervention. A retrospective cohort study of case notes of patients admitted with initial Glasgow Coma Scale 13–15 and injuries identified by CT was completed. Data on a primary outcome measure of clinically important deterioration (indicating need for hospital admission) and secondary outcome of neurosurgery, intensive care unit admission, or intubation (indicating need for neurosurgical admission) were collected. Multi-variable logistic regression was used to derive models and a risk score predicting deterioration using routinely reported clinical and radiological candidate variables identified in a systematic review. We compared the performance of this new risk score with the Brain Injury Guideline (BIG) criteria, derived in the United States. A total of 1699 patients were included from three English major trauma centers. A total of 27.7% (95% confidence interval [CI], 25.5–29.9) met the primary and 13.1% (95% CI, 11.6–14.8) met the secondary outcomes of deterioration. The derived clinical decision rule suggests that patients with simple skull fractures or intracranial bleeding <5 mm in diameter who are fully conscious could be safely discharged from the ED. The decision rule achieved a sensitivity of 99.5% (95% CI, 98.1–99.9) and specificity of 7.4% (95% CI, 6.0–9.1) to the primary outcome. The BIG criteria achieved the same sensitivity, but lower specificity (5%). Our empirical models showed good predictive performance and outperformed the BIG criteria. This would potentially allow ED discharge of 1 in 20 patients currently admitted for observation. However, prospective external validation and economic evaluation are required.

## Introduction

Over 1.4 million patients annually attend emergency departments (EDs) in the UK following head injury, of which 95% have a normal or mildly impaired conscious level at presentation—Glasgow Coma Scale (GCS) score of 13–15.^1^ The majority of ED computed tomography (CT) scans for diagnosing TBI are conducted in these patients with apparently mild injury. In this group, the prevalence of brain injuries, skull fractures, and intracranial bleeding is 7%, while only 1% of CT scans identify life-threatening TBI.^2^

The management of patients with mild TBI and injuries identified by CT imaging is controversial. Some centers advocate that all patients should be admitted under specialist neurosurgical care and undergo repeat CT imaging.^3,4^ The Brain Injury Guideline (BIG) criteria, a consensus-derived risk tool currently used in some centers in the United States, advocate the discharge of selected GCS 13–15 patients from the ED with injuries on CT (Supplementary Material 1).^5^ We recently published a systematic review of predictors of deterioration in this cohort identifying some single factors associated with deterioration, but there was no good empirical evidence to guide post imaging management in this group.^4^

In England, national (National Institute of Health and Clinical Excellence) TBI guidelines recommend that patients with TBI identified by CT are admitted to the hospital.^1^ However, they do not define which injuries are clinically significant and which patients benefit from specialist neurosurgical care. Other guidelines used internationally also recommend routine hospital admission for this group.^4^

There has been a paucity of research to inform the admission and referral decisions for these TBI patients with apparently mild injuries, but abnormalities on CT scan.^6^ Prediction modeling may help identify low-risk patients who could be safely discharged from the ED. Modeling may also facilitate earlier identification of patients requiring neurosurgical intervention.

The study aims were to:

1. Estimate the prevalence of clinically important deterioration in GCS 13–15 patients with traumatic CT abnormalities.
2. Develop prediction models for patient deterioration that could be used to inform hospital admission and specialist referral.
3. Compare the performance of an empirically derived prediction model with the BIG criteria.

## Methods

### Study design

We conducted a retrospective cohort study using case-note review of TBI patients presenting to the ED between 2010 and 2017 at three major trauma centers in England: Hull University Teaching Hospital NHS Trust, Salford Royal NHS Foundation Trust, and Addenbrooke's Hospital (Cambridge University Hospitals NHS Foundation Trust). A detailed study protocol has previously been published.^6^ The study was conducted and is reported in accordance with international guidelines for prognostic research.^7^

### Study population

#### Population selection

Within each study center ED, CT brain scan requests and reports were screened to identify patients with traumatic findings presenting between 2010 and 2017. Patients were matched to case records and if meeting the inclusion criteria data were extracted on patient deterioration outcomes and candidate predictors (see below).

### Inclusion criteria

Patients ≥16 years of age with a presenting GCS 13–15 who attended the ED after acute TBI and had injuries reported on CT brain scan were included. The latter was defined as: skull fractures, extradural haemorrhage, subdural haemorrhage with an acute component, intracerebral haemorrhage, contusions, subarachnoid hemorrhage, and intraventricular hemorrhage. Intracerebral, intraventricular, and subarachnoid hemorrhages were considered traumatic in etiology when a mechanism of injury or injuries indicating trauma were recorded.

### Exclusion criteria

Patients were excluded where: a non-traumatic cause of intracranial hemorrhage was indicated, pre-existing CT abnormality prevented determining whether acute injury had occurred, and patients transferred from other hospitals.

### Outcomes

#### Primary outcome

Deterioration up to 30 days after ED attendance was used, which was a composite including: death attributable to TBI, neurosurgery, seizure, a drop in GCS >1, intensive care unit (ICU) admission for TBI, intubation, or hospital readmission for TBI. Where reason for death, ICU admission, or readmission was unknown, it was attributed to TBI deterioration.

#### Secondary outcome

A composite measure indicating need for neurosurgical specialist admission was used, including: neurosurgery, ICU admission for TBI, or intubation up to 30 days after ED attendance.

### Predictors

Pre-injury anticoagulant and -platelet therapy were combined in a variable with two categories: 1) no therapy and 2) use of either or both medications (exploratory multi-variable modeling indicated they had similar effect sizes). Comorbidity was measured using the trauma modified Charlson comorbidity index.^8^ Rockwood Frailty Scale scores were assigned to patients >50 years of age using information in the case notes and data collapsed into established categories.^9,10^

Supplementary Material 2 outlines how injuries described in written CT reports were categorized. Injury severity was coded using the Abbreviated Injury Scale (AIS), injury size, and presence of midline shift or mass effect. AIS codes were mapped to the Marshall classification using the method described by Lesko and colleagues and the description of midline shift.^11^ An additional category of severity of up to two injuries with a combined maximal diameter <5 mm was added. TBI severity, as measured by the Marshall classification,^11^ was assessed for inclusion in the final model alongside type of hemorrhage, contusion or skull fracture present, and total number of injuries. This allowed the independent predictive value of each of these components of the CT scan to be simultaneously assessed.

### Sample size

A sample size requirement of 2000 patients was calculated using an estimated prevalence of deterioration of 10%.^6^ Interim analysis found the actual prevalence of deterioration to be around 25%. Therefore, the target was revised to 1700 patients, equating to 425 events and allowing 42 candidate factors to be assessed on the basis of 10 events per factor.^12^

### Statistical analysis

#### Model selection

The primary and secondary outcomes of deterioration were modelled as binary variables using logistic regression.^13^ We used stepwise selection to find the smallest number of candidate explanatory variables that accurately predict deterioration. Tables 1 and 2 summarize how candidate variables were included in modeling. For each candidate predictor, an unadjusted odds ratio was calculated.

**Table 1.** Characteristics of the Study Population

Candidate factor	Category	Mean (SD), min-max	Missing data
		OR* N *(%)	*N* = 1699
Age	Years	58.2 (SD 23.3)	None
		16–101	
		Age ≥65 = 44.9%	
Sex	Male	67% (median age = 52)	None
	Female	33% (median age = 69)	
GCS	15	976 (58%)	5 (0.3%)
	14	533 (31%)	
	13	185 (11%)	
Mechanism of injury	Assault	228 (13%)	31 (1.8%)
	Fall	1090 (64%)	
	Fall from height	361 (21%)	
	RTC	298 (18%)	
	Sport	21 (1%)	
	Other	30 (2%)	
Intoxicated	Yes	494 (29%)	38 (2.2%)
Seizure pre-hospital or in ED	Yes	74 (4%)	10 (0.6%)
Vomit pre-hospital or in ED	Yes	310 (18%)	12 (0.7%)
Preinjury anticoagulation or antiplatelets	Anticoagulation use	155 (9%)	None
	Antiplatelet use	294 (17.3%)	
	Both	8 (0.5%)	
Abnormal first neurological examination	Yes	233 (14.5%)	89 (5.2%)
Initial blood pressure	Mean arterial pressure mm Hg	98.5 (SD 17)	61 (3.6%)
		43–193	
Initial oxygen saturation	%	97.4 (SD 2.4)	59 (3.5%)
		80–100	
Initial respiratory rate	RR per min	17.9 (SD 3.5)	94 (5.5%)
		10–48	
Haemoglobin	g/L	136 (SD 19.1)	211 (12.4%)
		68–265	
Platelet value	10^9^/L	232 (SD 77)	211 (12.4%)
		2–742	
No. of injuries on CT	1	824 (48.5%)	None
	2	400 (23.6%)	
	3	217 (12.7%)	
	4	142 (8.4%)	
	5	103 (6.1%)	
	Multiple diffuse injury^a^	13 (0.8%)	
Injury severity on CT (Based on the Marshall classification system and described in detail in Supplementary Material 2)	1) Simple skull fractures	66 (3.9%)	None
	2) Complex skull fractures	123 (7.2%)	
	3) 1–2 bleeds <5 mm (total)	208 (12.2%)	
	4) No or minimal mass effect	1001 (58.9%)	
	5) Significant midline shift	159 (9.4%)	
	6) High/mixed-density lesion^b^	122 (7.2%)	
	7) Cerebellar/brainstem injury	22 (1.2%)	
Skull fracture (simple)	Yes	316 (19%)	None
Skull fracture (complex)	Yes	360 (21%)	None
Contusion	Yes	580 (34%)	None
Extradural bleed	Yes	135 (8%)	None
Intraparenchymal hemorrhage	Yes	240 (14%)	None
Subdural bleed	Yes	694 (41%)	None
Intraventricular bleed	Yes	50 (3%)	None
Subarachnoid bleed	Yes	536 (32%)	None
Rockwood Clinical Frailty Scale (CFS)	Patients under 50	649 (39%)	28 (1.6%) cases
	CFS 1–3	642 (38%)	
	CFS 4–6	308 (18.5%)	
	CFS 6–9	72 (4.5%)	
Comorbidity	Charlson index	1.4 (SD 2.9)	20 (1.2%) cases
		0–28 (range)	
ISS	Body regions excluding head	5.2 (SD 5.2)	None
		0–75 (range)	

^a^ Diffuse injuries refer to multiple tiny intracerebral hemorrhages/contusions/diffuse axonal injuries.

^b^ This category corresponds to Marshall Classification VI (volume >25 mL) and corresponds to a need for surgical evacuation by the Marshall Classification.

GCS, Glasgow Coma Scale; ED, emergency department; CT, computed tomography; ISS, Injury Severity Score; RTC, road traffic collision; RR, respiratory rate; SD, standard deviation; min-max, minimum/maximum; OR, odds ratio.

**Table 2.** Candidate Factors’ (Uni- and Multi-Variable) Associations with the Outcome of Deterioration

Candidate factor	Category	Univariable effect on risk of deterioration: odds ratio (95% CI)	Multi-variable effect on risk of deterioration: odds ratio (95% CI)
GCS versus 15	GCS 14	1.8 (1.4–2.3)	1.6 (1.2–2.1)
	GCS 13	3.1 (2.3–4.4)	2.3 (1.6–3.3)
Pre-injury anticoagulation or antiplatelets	Yes	1.7 (1.3–2.1)	1.4 (1.03–1.80)
Abnormal neurological examination	Abnormal	2.3 (1.7–3.0)	1.7 (1.2–2.3)
Hemoglobin	g/L (1-unit increase)	0.99 (0.98–0.99)	0.99 (0.98–1.00)
No. of injuries on CT versus 1	2	1.4 (1.1–1.9)	1.3 (0.97–1.80)
	3	1.8 (1.3–2.5)	1.6 (1.1–2.3)
	4	3.2 (2.2–4.7)	2.5 (1.6–3.8)
	5	3.7 (2.5–5.7)	2.8 (1.7–4.6)
	Diffuse injury	1.1 (0.3–4.2)	1.4 (0.3–5.3)
Injury severity on CT versus simple skull fracture (categories described in detail in Supplementary Material 2)	2) Complex skull fractures	1.4 (0.5–4.2)	1.4 (0.5–4.3)
	3) 1–2 bleeds <5 mm (total)	1.4 (0.5–3.8)	1.1 (0.4–3.1)
	4) No or minimal mass effect	4 (1.6–10.0)	2.3 (0.9–5.9)
	5) Significant midline shift	13.7 (5.2–35.8)	6.8 (2.5–18.5)
	6) High/mixed-density lesion	40.1 (15.0–111.9)	21.6 (7.7–60.7)
	7) Cerebellar/brainstem injury	8.1 (2.3–29.2)	7 (1.9–25.7)
Extracranial injury	ISS 1-unit increase	1.02 (1.00–1.04)	1.03 (1.002–1.050)
Age	Year 1-unit increase	1.01 (1.006–1.015)	^a^
Sex	Female	1.04 (0.83–1.31)	^a^
Intoxicated	Yes	0.98 (0.77–1.24)	^a^
Seizure pre-hospital or in ED	Yes	1.2 (0.7–2.0)	^a^
Vomit pre-hospital or in ED	Yes	1.3 (1.0–1.7)	^a^
Initial blood pressure	1-unit increase, mean arterial pressure mm Hg	1.004 (1.00–1.01)	^a^
Initial oxygen saturation	% (1-unit increase)	0.99 (0.95–1.04)	^a^
Initial respiratory rate	RR per min (1-unit increase)	1.05 (1.02–1.08)	^a^
Platelet value	10^9^/L (1-unit increase)	1 (0.997–1.000)	^a^
Skull fracture (simple)	Yes	1.1 (0.8–1.4)	^a^
Skull fracture (complex)	Yes	0.955 (0.7–1.2)	^a^
Contusion present	Yes	1.4 (1.1–1.7)	^a^
Extradural bleed	Yes	2 (1.4–2.9)	^a^
Intraparenchymal hemorrhage present	Yes	1.2 (0.9–1.6)	^a^
Subdural bleed	Yes	2.2 (1.8–2.8)	^a^
Intraventricular bleed	Yes	1.9 (1.81–3.40)	^a^
Subarachnoid bleed	Yes	1.4 (1.1–1.7)	^a^
Comorbidity	Charlson index	1.07 (1.03–1.11)	^a^
Rockwood Frailty Score versus under 50	CFS 1–3	1.3 (1.04–1.70)	^a^
	CFS 4–6	1.6 (1.2–2.2)	
	CFS 7–9	2.8 (1.7–4.6)	

^a^ Not selected into model.

GCS, Glasgow Coma Scale; CT, computed tomography; ED, emergency department; ISS, Injury Severity Score; RR, respiratory rate; CFS, Clinical Frailty Scale; CI, confidence interval.

The extent of missing data on each candidate variable is shown in Table 1. Where medication use was undocumented, it was taken to indicate no pre-injury use. For other variables, we assumed missing data occurred at random. Twenty-five imputed data sets were created (based on missing data in around 25% of cases) using chained equations including all candidate variables and outcomes in the ICE STATA package (StataCorp LP, College Station, TX).^14^ The midiagplots STATA function was used to compare the distributions of observed and imputed data.^15^ Where continuous variables were non-normally distributed and implausible, imputed values were generated; predictive mean matching was used.^14^

Model selection was performed using multi-variable backward elimination with a statistical significance threshold of 0.1. All candidate predictors were initially included and imputed data sets combined using Rubin's rules at each stage of model selection. For candidate continuous variables, rather than assume a linear relationships, the best predictive form was explored with the MFPMI function using backward elimination for fractional polynomial functions in multi-variable modeling.^16,17^ Fractional polynomials were limited to 2 degrees of freedom when predicting the secondary outcome.

#### Model performance

Model fit was assessed using the Briers score averaged across imputed data sets.^18^ A score of 0 implies perfect prediction and 0.25 no predictive value.

Model discrimination (how well patients with and without deterioration were distinguished) was assessed by the C-statistic, measured by combing estimates across imputed data sets using Rubin's rules.^17,19^

Calibration measures how well predictions made by models match observations.^13^ The calibration slope of selected predictors was calculated in each imputed data set and averaged.

#### Sensitivity analysis

Model selection and evaluation of model performance were repeated in patients with complete data.

#### Internal validation

Models tend to perform better on data from which they are derived (overfitting).^13^ Bootstrap internal validation with 100 bootstrap samples was performed in each imputed data set to calculate the average optimism. Model selection was repeated in each bootstrap sample, and performance of models selected was subtracted by performance in the original data set.^20, 21^ The pooled average difference in the calibration slope between the bootstrap samples and original data was averaged across imputed data sets. This was subtracted from the original averaged calibration slope to estimate the shrinkage factor. The shrinkage factor was applied to the derived model coefficients to adjust for optimism.^13^ The C statistic was adjusted for optimism using the same method.

#### Mild traumatic brain injury risk score development and comparison to the Brain Injury Guideline criteria

To use our prognostic model for making clinical decisions, we derived a risk score using optimism-adjusted coefficients.^22^ To make the risk score clinically interpretable, coefficients were standardised and rounded.^22^ Individual patient risk scores were calculated. A risk score for ED discharge was proposed based on the trade-off between risk of deterioration in a discharged patient and number of patients admitted for observation.

Sensitivity and specificity of the proposed discharge score and of the BIG criteria to deterioration were calculated and compared in patients with complete data for both criteria.

### Ethics

NHS Research Ethics Committee Approval was granted by West of Scotland REC 4 reference: 17/WS/0204. As a retrospective case review conducted by members of the direct care team, consent was not required.

## Results

### Study population

**FIG. 1.**
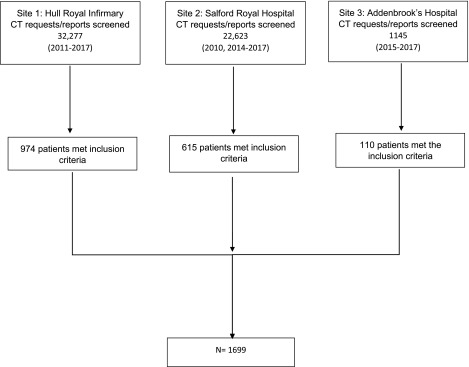
Population selection. CT, computed tomography.

Figure 1 summarizes study population selection and Table 1 population characteristics and candidate variables. The cohort was mostly male, with around half of patients >60 years of age and one quarter with either pre-injury anticoagulant or -platelet use. A total of 470 patients (27.7%; 95% confidence interval [CI], 25.5–29.9) clinically deteriorated as defined by the primary outcome. A total of 223 patients (13.1%; 95% CI, 11.6–14.8) underwent neurosurgery were admitted to ICU or were intubated (secondary outcome). A total of 72 patients had deaths attributable to TBI. A total of 471 patients had data missing from at least one candidate variable.

### Model selection

Table 2 summarizes the univariable associations between candidate variables and the primary outcome. Supplementary Material 3 presents the distributions of imputed data.

The equivalent of 41 candidate factors were assessed in multi-variable modeling to predict patient deterioration, and 34 factors were assessed in modeling to predict need for neurosurgical referral. The selected model predicting the primary outcome is presented in Table 2 and the secondary outcome in Table 3. Supplementary Material 4 presents a complete case sensitivity analysis.

**Table 3.** Candidate Factors’ (Uni- and Multi-Variable) Association with Neurosurgical Admission

Candidate factor	Category	Univariable effect on risk of deterioration: odds ratio (95% CI)	Multi-variable effect on risk of deterioration: odds ratio (95% CI)	
Age	Year (1-unit increase)	0.99 (0.99–1.00)	(Age/10)^3^	0.997 (0.9960–0.9989)
GCS versus 15	GCS 14	2 (1.5–2.8)	2.3 (1.6–3.3)	
	GCS 13	3.8 (2.6–5.7)	3.7 (2.3–5.9)	
Abnormal neurological examination	Abnormal	2.4 (1.7–3.4)	1.9 (1.3–3.0)	
Hemoglobin	g/L (1-unit increase)	1 (0.99–1.01)	0.99 (0.98–1.00)	
Injury severity on CT versus simple skull fracture (categories described in detail in Supplementary Material 2)	2) Complex skull fractures	1.9 (0.4–9.6)	0.9 (0.5–4.9)	
	3) 1–2 bleeds <5 mm (total)	1 (0.2–4.8)	0.8 (0.1–4.1)	
	4) No or minimal mass effect	3.3 (0.8–13.6)	2.3 (0.5–9.7)	
	5) Significant midline shift	11.5 (2.7–49.0)	7.4 (1.6–33.9)	
	6) High/mixed-density lesion	41.7 (9.8–178.0)	37.1 (8.1–169.0)	
	7) Cerebellar/brainstem injury	8 (1.3–47.6)	8.5 (1.3–56.2)	
Skull fracture (complex)	Yes	1.7 (1.3–2.3)	2 (1.3–3.0)	
Subdural bleed	Yes	2.2 (1.6–2.9)	1.7 (1.2–2.5)	
Extracranial onjury	ISS (1-unit increase)	1.03 (1.004–1.060)	1.06 (1.03–1.09)	
Rockwood Frailty Score versus under 50	CFS 1–3	1.2 (0.9–1.6)	1.9 (1.1–3.1)	
	CFS 4–6	0.4 (0.2–0.7)	0.7 (0.3–1.8)	
	CFS 7–9	0.09 (0.01–0.60)	0.09 (0.01–0.70)	
Sex	Female	0.66 (0.48–0.91)	^a^	
Preinjury anticoagulation or antiplatelets	Yes	0.95 (0.7–1.3)	^a^	
Intoxicated	Yes	1.1 (0.8–1.5)	^a^	
Seizure pre-hospital or in ED	Yes	1.8 (0.99–3.18)	^a^	
Vomit pre-hospital or in ED	Yes	1.5 (1.1–2.1)	^a^	
Initial blood pressure	1 unit increase, mean arterial pressure mm Hg	1.006 (1.00–1.01)	^a^	
Initial oxygen saturation	% (1-unit increase)	1 (0.94–1.07)	^a^	
Initial respiratory rate	RR per min (1-unit increase)	1 (0.99–1.07)	^a^	
Platelet value	10^9^/L (1-unit increase)	0.99 (0.998–1.001)	^a^	
Number of injuries on CT versus 1	2	1.4 (0.98–2.10)	^a^	
	3	1.5 (1.0–2.4)		
	4	3.4 (2.2–5.3)		
	5	4.3 (2.7–7.0)		
	Diffuse injury	1.8 (0.4–8.3)		
Skull fracture (simple)	Yes	1.2 (0.8–1.7)	^a^	
Contusion present	Yes	1.3 (0.997–1.800)	^a^	
Extradural bleed	Yes	2.6 (1.7–3.9)	^a^	
Intraparenchymal hemorrhage present	Yes	0.7 (0.5–1.2)	^a^	
Intraventricular bleed	Yes	0.7 (0.3–1.9)	^a^	
Subarachnoid bleed	Yes	1.4 (1.0–1.9)	^a^	
Comorbidity	Charlson index (1-unit increase)	0.94 (0.89–1.00)	^a^	

^a^ Not selected into model.

GCS, Glasgow Coma Scale; CT, computed tomography; ED, emergency department; ISS, Injury Severity Score; CFS, Clinical Frailty Scale; RR, respiratory rate; CI, confidence interval.

### Model performance

Table 4 summarizes measures of model performance. The models predicting the primary and secondary outcomes had Briers scores of 0.16 and 0.09, respectively. The model predicting composite deterioration (primary outcome) had an optimism-adjusted C-statistic of 0.75, and the model predicting need for specialist neurosurgical admission had an optimism-adjusted C-statistic of 0.85. The trade-off between the sensitivity and specificity of these models is shown in the receiver operating characteristic curves in Supplementary Material 5.

**Table 4.** Performance of Predictive Models

Outcome	Measure	Apparent performance	Average optimism	Optimism adjusted
Clinical deterioration	Brier score	0.16		
	Calibration slope	1	0.14	0.86
	C-statistic	0.773	0.026	0.747
Need for specialist neurosurgical admission	Brier Score	0.09		
	Calibration slope	1	0.04	0.96
	C-statistic	0.86	0.01	0.85

### The mild traumatic brain injury risk score

Table 5 presents the weighted risk score derived from our prognostic model predicting deterioration. Hemoglobin, although a statistically significant predictor in multi-variable modeling, was not included given that, because of the small effect size and range of abnormal values, inclusion did not improve performance (Supplementary Material 6). Based on the trade-off between sensitivity and specificity, a patient risk score of 0 was used as a threshold for ED discharge. Patients at this cutoff had the following characteristics: initial GCS 15, single simple skull fracture or hemorrhage <5 mm, up to two extracranial bony or organ injuries not requiring hospital admission, not anticoagulated/taking antiplatelets, no cerebellar/brain stem injuries, and normal neurological examination (Table 5). Patients with a risk score of 1–5 had a 17.5% risk of deterioration, and patients with a risk score >5 had 54% risk of deterioration (Supplementary Material 7).

**Table 5.** Mild TBI Risk Score

Factor	Coefficient (optimism adjusted)	Risk score value
Pre-injury anticoagulation or antiplatelets	0.3	1
GCS		
15	0 (vs.)	**GCS 15** 0
14	0.4	**GCS 14** 1
13	0.7	**GCS 13** 2
Normal first neurological examination	0.45	**Abnormal** 1.5
No. of Injuries on CT		
**1**	0 (vs.)	**1** 0
**2**	0.25	**2** 1
**3**	0.4	**3** 1
**4**	0.8	**4** 3
**5**	0.9	**5** 3
**Diffuse**	0.3	**Diffuse** 1
Injury severity on CT^a^		
**1** simple skull fracture	0 (vs.)	**1** 0
**2** complex skull fracture	0.3	**2** 1
**3** 1–2 bleeds <5 mm	0.08	**3** 0
**4** No or minimal mass effect	0.7	**4** 2
**5** Significant midline shift	1.7	**5** 5
**6** High/mixed-density lesion	2.7	**6** 9
**7** Cerebellar/brainstem injury	1.7	**7** 5
ISS (body regions excluding head)	0.2	**Up to 2 non-significant extra-cranial injuries^b^** 0
		**Any significant extracranial injury or ≥3 injuries** 2
Hb	–0.01	Not included in risk score
Constant	–1.38	

^a^ TBI severity categories are described in detail in Supplementary Material 2.

^b^ Injuries exclude superficial lacerations and abrasions, and a significant extracranial injury is defined as any injury requiring inpatient care.

TBI, traumatic brain injury; GCS, Glasgow Coma Scale; CT, computed tomography; ISS, Injury Severity Score; Hb, hemoglobin.

The performance of the BIG criteria and our risk score were assessed in the 1569 patients with complete data for both classification systems. A threshold of 0 in our risk score achieved a sensitivity of 99.5% (95% CI, 98.1–99.9) and specificity of 7.4% (95% CI, 6.0–9.1) to the primary outcome. The BIG criteria for discharge achieved the same sensitivity for deterioration, but lower specificity, although the confidence intervals overlap and this may be due to chance. Table 6 summarizes the characteristics of the false negatives (patients meeting the discharge threshold who deteriorated) in both approaches. No patients recommended for discharge by either criteria died or required neurosurgery, but 1 patient recommended for discharge by the BIG criteria required intubation. The BIG criteria would have allowed discharge of 57 patients (3.6%) compared to 87 patients (5.5%) with our risk score.

**Table 6.** Performance of mTBI Risk Score and BIG Criteria

*N* = 1569	Deteriorated	Did not deteriorate	Positive predictive value (PPV)
			Negative predictive value (NPV)
Performance of risk score			
Admission (score >0)	423	1059	PPV = 28.5%
Discharge (score = ≤0)	2^a^	85	NPV = 97.7%
	Sensitivity = 99.5%	Specificity = 7.4%	
	(95% CI, 98.1–99.9)	(95% CI, 6.0–9.1)	
Performance of BIG criteria			
Admit (not BIG1)	423	1089	PPV = 28%
Discharge (BIG 1)	2^a^	55	NPV = 96.5%
	Sensitivity = 99.5%	Specificity = 4.8%	
	(95% CI, 98.1–99.9)	(95% CI, 3.7–6.3)	

^a^ Patients recommended for discharge by our risk score who deteriorated:

1) 85 female, small subdural dropped GCS. Rockwood Frailty Score, 4.

2) 56 male, small contusion (report stated possible second small intracranial hemorrhage, only first injury included) and pre-injury seizure. Seizure during admission.

Patients triaged to discharge by BIG who deteriorated:

1) 85 female, small subdural dropped GCS. Rockwood Frailty Score, 4.

2) 55 female, small subdural and polytrauma (ISS 10). Required intubation.

mTBI, mild traumatic brain injury; BIG, Brain Injury Guideline; CI, confidence interval; GCS, Glasgow Coma Scale; ISS, Injury Severity Score.

## Discussion

### Summary

To our knowledge, this is the first UK study to report the risk of deterioration in all initial mild TBI patients with traumatic injuries reported on CT brain scan and study internationally to develop a prognostic model and risk tool for avoiding unnecessary hospital admissions. We also report the first independent validation of the BIG criteria.

The estimated prevalence of deterioration was 27.7%. Our prognostic models for composite measures of deterioration had optimism adjusted C statistics of 0.75 and 0.85, indicating good discrimination between patients with and without deterioration or need for neurosurgical care.

Using our risk score, derived from the prognostic model, to hypothetically direct need for hospital admissions we identified that it would appear safe to discharge from the ED patients who are fully conscious with no focal neurology (GCS 15)—not taking anticoagulant or -iplatelet medication who have a single simple skull fracture or hemorrhage <5mm (not cerebellar or brainstem) on CT brain scan and up to two extracranial bony or organ injuries not requiring hospital admission (risk score 0). This derived decision rule achieved a sensitivity of 99.5% and specificity of 7.4% for deterioration. Categorization of patients for discharge using the BIG criteria achieved the same sensitivity, but a lower specificity.

The model predicting need for neurosurgical admission (based on risk of an interventional outcome) found higher age and frailty reduces risk. This probably reflects clinical selection of patients, with frail older patients less likely to undergo invasive interventions.

### Strengths

We believe this is the largest multi-center cohort study undertaken to estimate the prevalence of a composite measure of deterioration in this population.^4^ The study was powered to develop a prognostic model predicting this outcome. Candidate predictor factors were selected *a priori* on the basis of existing literature.^6^ We followed established techniques for handling missing data, prognostic modeling, and adjusting for optimism.^7,13,16,23^ Unlike risk stratification systems based solely upon CT findings,^24–26^ we have assessed a range of additional patient characteristics, test results, and other clinical factors for deterioration for inclusion in our model so as to achieve the maximum predictive accuracy. Our risk score is the first empirically derived scoring system which can to be used to inform admission decisions in this TBI population and incorporates both patient characteristics and other clinical risk factors alongside CT findings.

### Limitations

Because of the resource implications of conducting a prospective study, we pragmatically chose a retrospective study design. Around 25% of patients had missing data, but given that these data were mainly missing through poor recording or missing notes, and therefore missing at random, imputation techniques were valid. Documentation inaccuracies may have introduced random error, but are unlikely to have introduced systematic bias.

We classified TBI severity using information in written CT reports by using AIS coding to map to a modified Marshall classification. Poor reporting of the size of injuries and extent of mass effect meant most injuries were classified as equivalent to Marshall classification II. Better systematic and standardized reporting may have allowed TBI severity to be better classified and improved the performance of the derived models. We were unable to assess whether using other scoring systems to classify TBI severity, such as the Stockholm, Helsinki, or NeuroImaging Radiological Interpretation System scoring systems, would improve the performance of the derived model.^24–26^ Unlike with the Marshall classification, there is no validated way to map between AIS coding and these classification systems. However, type of injury was considered for inclusion in the model, alongside the Marshall classification and number of injuries.

Outcomes were limited to those recorded in hospital records, which may mean that patient deterioration in the community was missed. However, this is unlikely, and a check in Hull of deaths recorded in patients eligible for entry on the national trauma registry (linked to the office of national statistic mortality reporting) found no missed deaths.

We only assessed the predictive value of routinely collected factors. We could not assess the potential predictive value of using non-routinely collected variables identified in our review^6^ or biomarkers.

Although we have internally validated our derived models, they have not been externally validated. There is debate about the best way to combine imputation of missing data and internal validation bootstrapping techniques.^21^ We chose to bootstrap within imputations because of lower computational complexity. This has been shown, in simulation studies, to provide accurate estimates of the shrinkage factor.^21^ Other studies^27^ found imputing within bootstraps better adjusts for optimism, and therefore, despite adjusting for overfitting, our models may perform less well when applied to new data.

The lower prevalence of the secondary outcome than expected means our study may not be adequately powered to derive a model accurately predicting this outcome.

### Comparison previous literature

The estimated prevalence of clinical deterioration at 27.7% was higher than previously reported. In our review, we found the pooled prevalence of clinical deterioration to be around 10%.^4^ This reflects differences in study design; previous studies used narrower outcome definitions, such as neurological deterioration or ICU intervention,^4^ while we used a wide composite primary outcome aimed at encompassing need for hospital admission. We assessed an unselected GCS 13–15 population, while previous studies often restricted their inclusion criteria on the basis of GCS scores, injury severity, admitting inpatient specialty, and medication use.^6^

Research assessing prognostic factors in this TBI population have frequently used sample sizes based on convenience and lacked the statistical power to assess potential predictors simultaneously.^4,28^ Our study was sufficiently powered to assess over 40 candidate variables in multi-variable modeling. Previous research found that initial GCS, type of brain injury, anticoagulation, and age were the strongest predictors of adverse outcomes in this population.^4^ In our multi-variable model, all these factors were also found to be predictors of deterioration.

Studies evaluating the BIG criteria in the level 1 trauma center in the United States, where it is routinely applied, found that around 10% of patients met the criteria for ED discharge and no patient that met these criteria had adverse outcomes.^5,29^ In our cohort, 4% of patients met the criteria for ED discharge and 2 of these patients deteriorated. Our study cohort was, on average, older and had a lower GCS than studies previously assessing the BIG criteria, which may account for the difference in performance.

### Implications

Internationally, and particularly in the United States, there is wide variation in admission practices in this group with a range of specialist admission and discharge criteria used on the basis of limited evidence.^5,30–32^ Accurate risk prediction has the potential to help rationalize admission decisions in this group. Between April 2014 and June 2015, around 11,000 TBI patients were admitted to specialist neurosurgical centers in the UK and over 50% of these patients had mTBI.^33^ Currently, all patients with TBI identified by CT imaging are admitted to the hospital. Therefore, despite the low specificity of our model and the high false-positive rate, application of our model could improve clinical care by reducing unnecessary hospital admissions and thereby save health service resources and reduce patient inconvenience.

Our risk tool demonstrated good predictive sensitivity (99.5%) to our primary outcome at the proposed threshold for ED discharge. This would have allowed the discharge of 87 of 1569 patients (5.5%). At this sensitivity, a negative predictive value of 97.7% was achieved (an approximately 1 in 50 chance of a discharged patient deteriorating). This may not be clinically acceptable, but no patient recommended by our risk score for discharge died or required neurosurgery or an ICU intervention. One patient recommended for discharge had a report indicating a possible second lesion and therefore may have been admitted in clinical practice. The BIG criteria achieved the same sensitivity (99.5%) to the primary outcome, but its lower specificity means that clinical application would result in fewer patients being discharged.

The high predictive accuracy of our model for the secondary outcome (area under the curve = 0.85) suggests that it could be used to inform neurosurgical admissions in this population. The acceptable level of risk of requiring invasive intervention for a patient admitted under a non-specialist team is unknown and is likely to vary between centers. The lower prevalence of this outcome means that the estimated model may be less accurate, and we regard this as a starting point for further research.

Both our prognostic model and the BIG criteria should be validated prospectively before they could be used in clinical practice. A prospective study design would address the weaknesses in outcome collection highlighted earlier, including assessing the predictive value of CT severity classification systems other than the Marshall classification system, and allow the inclusion of non-routinely collected prognostic factors, including biomarkers. Improved systematic reporting of CT scans could possibly increase the predictive accuracy of our model and further increase the performance of our risk tool.^25,34^ Economic evaluation is also required to comprehensively assess the implication for both patient outcomes and resource use of using the model.

## Conclusion

This is the first study to empirically derive a prognostic model for patients with mTBI and injuries identified by CT imaging and independently validate the BIG criteria. Our empirically derived risk tool performed better than the BIG criteria and could be used to safely discharge from the ED 1 in 20 patients currently routinely admitted for observation. Both our prognostic model and the BIG criteria now require prospective external validation and economic evaluation.

## Supplementary Material

Supplemental data

## References

[1] National Institute for Health and Care Excellence (NICE). (2014). National Clinical Guidance Centre. (2014). CG 176 Head Injury Triage, assessment, investigation and early management of head injury in children, young people and adults. National Institute for Health and Care Excellence, Department of Health: London 25340248

[2] Haydel M.J., Preston C.A., Mills T.J., Luber S., Blaudeau E., and DeBlieux P.M. (2000). Indications for computed tomography in patients with minor head injury. N. Engl. J. Med. 343, 100–105 10891517 10.1056/NEJM200007133430204

[3] Thomas B.W., Mejia V.A., Maxwell R.A., Dart B.W., Smith P.W., Gallagher M.R., Claar S.C., Greer S.H., and Barker D.E. (2010). Scheduled repeat CT scanning for traumatic brain injury remains important in assessing head injury progression. J. Am. Coll. Surg. 210, 824–830, 831–822 20421059 10.1016/j.jamcollsurg.2009.12.039

[4] Marincowitz C., Lecky F.E., Townend W., Borakati A., Fabbri A., and Sheldon T.A. (2018). The risk of deterioration in GCS13–15 patients with traumatic brain injury identified by computed tomography imaging: a systematic review and meta-analysis. J. Neurotrauma 35, 703–718 29324173 10.1089/neu.2017.5259 PMC5831640

[5] Joseph B., Friese R.S., Sadoun M., Aziz H., Kulvatunyou N., Pandit V., Wynne J., Tang A., O'Keeffe T., and Rhee P. (2014). The BIG (Brain Injury Guidelines) project: defining the management of traumatic brain injury by acute care surgeons. J. Trauma Acute Care Surg. 76, 965–969 24662858 10.1097/TA.0000000000000161

[6] Marincowitz C., Lecky F.E., Townend W., Allgar V., Fabbri A., and Sheldon T.A. (2018). A protocol for the development of a prediction model in mild traumatic brain injury with CT scan abnormality: which patients are safe for discharge? Diagn. Progn. Res. 2, 6 31093556 10.1186/s41512-018-0027-4 PMC6460841

[7] Moons K.G., Altman D.G., Reitsma J.B., Ioannidis J.P., Macaskill P., Steyerberg E.W., Vickers A.J., Ransohoff D.F., and Collins G.S. (2015). Transparent Reporting of a multivariable prediction model for Individual Prognosis or Diagnosis (TRIPOD): explanation and elaboration. Ann. Intern. Med. 162, W1–73 25560730 10.7326/M14-0698

[8] Bouamra O., Jacques R., Edwards A., Yates D.W., Lawrence T., Jenks T., Woodford M., and Lecky F. (2015). Prediction modelling for trauma using comorbidity and ‘true’ 30-day outcome. Emerg. Med. J 32, 933–938 26493123 10.1136/emermed-2015-205176

[9] Gregorevic K.J., Hubbard R.E., Lim W.K., and Katz B. (2016). The clinical frailty scale predicts functional decline and mortality when used by junior medical staff: a prospective cohort study. BMC Geriatr. 16, 117 27250650 10.1186/s12877-016-0292-4 PMC4890513

[10] Rockwood K., Song X., MacKnight C., Bergman H., Hogan D.B., McDowell I., and Mitnitski A. (2005). A global clinical measure of fitness and frailty in elderly people. CMAJ 173, 489–495 16129869 10.1503/cmaj.050051 PMC1188185

[11] Lesko M.M., Woodford M., White L., O'Brien S.J., Childs C., and Lecky F.E. (2010). Using Abbreviated Injury Scale (AIS) codes to classify computed tomography (CT) features in the Marshall System. BMC Med. Res. Methodol. 10, 72 20691038 10.1186/1471-2288-10-72 PMC2927606

[12] Peduzzi P., Concato J., Kemper E., Holford T.R., and Feinstein A.R. (1996). A simulation study of the number of events per variable in logistic regression analysis. J. Clin. Epidemiol. 49, 1373–1379 8970487 10.1016/s0895-4356(96)00236-3

[13] Steyerberg E.W., Harrell F.E. Jr., Borsboom G.J., Eijkemans M., Vergouwe Y., and Habbema J.D.F. (2001). Internal validation of predictive models: efficiency of some procedures for logistic regression analysis. J. Clin. Epidemiol. 54, 774–781 11470385 10.1016/s0895-4356(01)00341-9

[14] White I.R., Royston P., and Wood A.M. (2011). Multiple imputation using chained equations: issues and guidance for practice. Stat. Med. 30, 377–399 21225900 10.1002/sim.4067

[15] Eddings W., and Marchenko Y. (2012). Diagnostics for multiple imputation in Stata. Stata J. 12, 353–367

[16] Morris T.P., White I.R., Carpenter J.R., Stanworth S.J., and Royston P. (2015). Combining fractional polynomial model building with multiple imputation. Stat. Med. 34, 3298–3317 26095614 10.1002/sim.6553 PMC4871237

[17] Wood A.M., White I.R., and Royston P. (2008). How should variable selection be performed with multiply imputed data? Stat. Med. 27, 3227–3246 18203127 10.1002/sim.3177

[18] Rufibach K. (2010). Use of Brier score to assess binary predictions. J. Clin. Epidemiol. 63, 938–939 20189763 10.1016/j.jclinepi.2009.11.009

[19] Cook N.R. (2007). Use and misuse of the receiver operating characteristic curve in risk prediction. Circulation 115, 928–935 17309939 10.1161/CIRCULATIONAHA.106.672402

[20] Heymans M.W., van Buuren S., Knol D.L., van Mechelen W., and de Vet H.C. (2007). Variable selection under multiple imputation using the bootstrap in a prognostic study. BMC Medical research methodology 7, 33 17629912 10.1186/1471-2288-7-33 PMC1945032

[21] Schomaker M., and Heumann C. (2018). Bootstrap inference when using multiple imputation. Stat. Med. 37, 2252–2266 29682776 10.1002/sim.7654 PMC5986623

[22] Battle C., Hutchings H., Lovett S., Bouamra O., Jones S., Sen A., Gagg J., Robinson D., Hartford-Beynon J., Williams J., and Evans A. (2014). Predicting outcomes after blunt chest wall trauma: development and external validation of a new prognostic model. Crit. Care 18, R98 24887537 10.1186/cc13873 PMC4095687

[23] Morris T.P., White I.R., and Royston P. (2014). Tuning multiple imputation by predictive mean matching and local residual draws. BMC Med. Res. Methodol. 14, 75 24903709 10.1186/1471-2288-14-75 PMC4051964

[24] Olivecrona M., Olivecrona Z., and Koskinen L. (2016). The Stockholm Score for the prediction of outcome in persons with severe traumatic brain injury treated with an ICP-targeted therapy. J. Neurotrauma 33, A34-A34

[25] Wintermark M., Li Y., Ding V.Y., Xu Y., Jiang B., Ball R.L., Zeineh M., Gean A., and Sanelli P. (2018). Neuroimaging radiological interpretation system for acute traumatic brain injury. J. Neurotrauma 35, 2665–2672 29665763 10.1089/neu.2017.5311

[26] Raj R., Siironen J., B. Skrifvars M., Hernesniemi J., and Kivisaari R. (2014). Predicting outcome in traumatic brain injury: development of a novel computerized tomography classification system (Helsinki computerized tomography score). Neurosurgery 75, 632–647 25181434 10.1227/NEU.0000000000000533

[27] Wahl S., Boulesteix A.-L., Zierer A., Thorand B., and van de Wiel M.A. (2016). Assessment of predictive performance in incomplete data by combining internal validation and multiple imputation. BMC Med. Res. Methodol. 16, 144–144 27782817 10.1186/s12874-016-0239-7 PMC5080703

[28] Joseph B., Pandit V., Aziz H., Kulvatunyou N., Zangbar B., Green D.J., Haider A., Tang A., O'Keeffe T., Gries L., Friese R.S., and Rhee P. (2015). Mild traumatic brain injury defined by Glasgow Coma Scale: is it really mild? Brain Injury 29, 11–16 25111571 10.3109/02699052.2014.945959

[29] Joseph B., Aziz H., Pandit V., Kulvatunyou N., Sadoun M., Tang A., O'Keeffe T., Gries L., Green D.J., Friese R.S., Lemole M.G., and Rhee P. (2014). Prospective validation of the brain injury guidelines: Managing traumatic brain injury without neurosurgical consultation. J. Trauma Acute Care Surg. 77, 984–988 25423541 10.1097/TA.0000000000000428

[30] Kreitzer N., Lyons M.S., Hart K., Lindsell C.J., Chung S., Yick A., and Bonomo J. (2014). Repeat neuroimaging of mild traumatic brain-injured patients with acute traumatic intracranial hemorrhage: clinical outcomes and radiographic features. Acad. Emerg. Med. 21, 1084–1091 10.1111/acem.12479 PMC4283790 25308130

[31] Pruitt P., Penn J., Peak D., and Borczuk P. (2017). Identifying patients with mild traumatic intracranial hemorrhage at low risk of decompensation who are safe for ED observation. Am. J. Emerg. Med. 35, 255–259 27838043 10.1016/j.ajem.2016.10.064

[32] Schaller B., Evangelopoulos D.S., Muller C., Martinolli L., Pouljadoff M.P., Zimmermann H., and Exadaktylos A.K. (2010). Do we really need 24-h observation for patients with minimal brain injury and small intracranial bleeding? The Bernese Trauma Unit Protocol. Emerg. Med. J 27, 537–539 20360489 10.1136/emj.2009.073031

[33] Marincowitz C., Lecky F., Allgar V., and Sheldon T. (2019). Evaluation of the impact of the NICE head injury guidelines on inpatient mortality from traumatic brain injury: an interrupted time series analysis. BMJ Open 9, e028912 10.1136/bmjopen-2019-028912 PMC6561604 31167873

[34] Maas A.I., Menon D.K., Steyerberg E.W., Citerio G., Lecky F., Manley G.T., Hill S., Legrand V., and Sorgner, A.; CENTER-TBI Participants and Investigators. (2015). Collaborative European NeuroTrauma Effectiveness Research in Traumatic Brain Injury (CENTER-TBI): a prospective longitudinal observational study. Neurosurgery 76, 67–80 25525693 10.1227/NEU.0000000000000575

