# Supplementary material for: Development of a Clinical Decision Rule for the Early Safe Discharge of Patients with Mild Traumatic Brain Injury and Findings on Computed Tomography Brain Scan: A Retrospective Cohort Study
Source: J Neurotrauma. 2019 Dec 20;37(2):324–33. doi: 10.1089/neu.2019.6652 (PMC6964807; doi:10.1089/neu.2019.6652)
Supplement: Supplemental data [file Supp_Material.docx]

Supplementary Material 1: The Brain Injury Guideline (BIG) criteria:

|  | BIG1 (Discharge from ED after 6 hours) | BIG2 (Non-specialist hospital admission) | BIG3* (Specialist hospital admission) |
| --- | --- | --- | --- |
| Neurological Examination | GCS13-15  Normal pupils  No Focal Neurological deficit | GCS13-15  Normal pupils  No Focal Neurological deficit | GCS<13  Or Abnormal pupils  Or Focal Neurological deficit |
| Intoxicated | No | No/Yes | No/Yes |
| Anticoagulants or Anti-platelets | No | No | Yes |
| Skull Fracture | No | Non-displaced | Displaced |
| Intracranial Bleed | Subdural Haemorrhage <5mm Or  Extradural Haemorrhage <5mm  Or  1 Intraparenchymal Haemorrhage <5mm  Or Trace Subarachnoid Haemorrhage | Subdural Haemorrhage 5-7mm Or  Extradural Haemorrhage 5-7mm  Or  1-2 Intraparenchymal Haemorrhages 5-7mm  Or Localised Subarachnoid Haemorrhage | All other injuries |
| Intra-ventricular Haemorrhage | No | No | Yes |

*Patients must fulfil all the criteria of BIG1 or BIG2 to be categorised as such and are otherwise automatically in BIG3

Supplementary material 2: Categorisation of TBI severity

| Category | Injury Description written CT report | AIS Codes | Equivalent Marshal Classification (Lesko et at^11^) |
| --- | --- | --- | --- |
| 1 | Vault skull fractures | 150000, 150400 150402 |  |
| 2 | Basal, depressed, open skull fractures | 150200, 150204, 150205, 150206, 150404, 150406, 150408 | I |
| 3 | 1-2 Bleeds* /contusions total diameter <5mm | 140605, 140631, 140639, 140651, 140693, 140694 (and written CT report indicated injury <5mm) |  |
| 4 | Bleed/contusion  No or minor mass effect | 140602,140604,140606,140612,140614,140611,140620,140622, 140628,140629,140630,140632,140634,140638,140640,140642, 140644,140646,140650,140652,140654,140684,140688, 140686, 140699, 140676, 140678, 140680, 140682, 140799 | II |
| 5** | Bleed/contusion Significant midline shift or mass effect indicated in CT report | 140202, 140660, 140662, 140664, 140666 | III/IV |
| 6 | Non-evacuated mass lesion.  High or mixed density mass lesion*** | 140608,140610,140616,140618,140624,140626,140636,140648, 140656, 140637, 140655 | VI |
| 7 | Cerebellar/brainstem injury | 140204,140206,140208,140210,140212,140214,140218,140299,  140402,140403,140404,140405,140406,140410,140414,140418,  140422,140426,140430,140434,140438,140442,140446,140450,  140458,140462,140466,140470,140474,140499, | VII |

*Bleeds refers to subdural, extradural, intracerebral and subarachnoid haemorrhage

**Written CT reports did not allow easy differentiation in the extent of mass effect, and therefore Marshall III and IV categories were collapsed into 1 category.

***This category refers to any lesion or combination of lesions where the mass effect is so great that the Marshall Classification recommends immediate surgical intervention.

Supplementary material 3: Distribution of observed and imputed data of first 6 imputations of 25

Saturations:

Respiratory Rate:

Hb:

Platelets:

Charlson Score:

MAP:

Intoxication:

|  | Imputation 1 | Imputation 2 | Imputation 3 | Imputation 4 | Imputation 5 | Imputation 6 |
| --- | --- | --- | --- | --- | --- | --- |
| Observed | 29.7% | 29.7% | 29.7% | 29.7% | 29.7% | 29.7% |
| Imputed | 42.1% | 34.2% | 34.2% | 39.5% | 47.4% | 36.8% |
| Completed | 30% | 29.8% | 29.8% | 30% | 30.1% | 29.9% |

Prehospital or ED Seizure:

|  | Imputation 1 | Imputation 2 | Imputation 3 | Imputation 4 | Imputation 5 | Imputation 6 |
| --- | --- | --- | --- | --- | --- | --- |
| Observed | 4.4% | 4.4% | 4.4% | 4.4% | 4.4% | 4.4% |
| Imputed | 0% | 22.3% | 0% | 11.1% | 0% | 11.1% |
| Completed | 4.4% | 4.5% | 4.4% | 4.4% | 4.4% | 4.4% |

Prehospital or ED Vomiting:

|  | Imputation 1 | Imputation 2 | Imputation 3 | Imputation 4 | Imputation 5 | Imputation 6 |
| --- | --- | --- | --- | --- | --- | --- |
| Observed | 18.4% | 18.4% | 18.4% | 18.4% | 18.4% | 18.4% |
| Imputed | 8.3% | 16.7% | 16.7% | 16.7% | 33.3% | 25% |
| Completed | 18.3% | 18.4% | 18.4% | 18.4% | 18.5% | 18.4% |

GCS:

| GCS:15 | Imputation 1 | Imputation 2 | Imputation 3 | Imputation 4 | Imputation 5 | Imputation 6 |
| --- | --- | --- | --- | --- | --- | --- |
| Observed | 57.6% | 57.6% | 57.6% | 57.6% | 57.6% | 57.6% |
| Imputed | 60% | 40% | 60% | 60% | 80% | 40% |
| Completed | 57.6% | 57.6% | 57.6% | 57.6% | 57.6% | 57.6% |
| GCS:14 | Imputation 4 | Imputation 2 | Imputation 4 | Imputation 4 | Imputation 5 | Imputation 6 |
| Observed | 31.5% | 31.5% | 31.5% | 31.5% | 31.5% | 31.5% |
| Imputed | 40% | 40% | 40% | 40% | 20% | 60% |
| Completed | 31.5% | 31.5% | 31.5% | 31.5% | 31.5% | 31.5% |
| GCS:13 | Imputation 4 | Imputation 2 | Imputation 4 | Imputation 4 | Imputation 5 | Imputation 6 |
| Observed | 10.9% | 10.9% | 10.9% | 10.9% | 10.9% | 10.9% |
| Imputed | 0% | 20% | 0% | 0% | 0% | 0% |
| Completed | 10.9% | 10.9% | 10.9% | 10.0% | 10.9% | 10.0% |

Abnormal First Neurological Examination:

|  | Imputation 1 | Imputation 2 | Imputation 3 | Imputation 4 | Imputation 5 | Imputation 6 |
| --- | --- | --- | --- | --- | --- | --- |
| Observed | 14.5% | 14.5% | 14.5% | 14.5% | 14.5% | 14.5% |
| Imputed | 14.6% | 30.3% | 21.3% | 21.3% | 19.1% | 13.5% |
| Completed | 14.5% | 15.3% | 14.8% | 14.8% | 14.7% | 14.4% |

Frailty (no missing data under 50 category):

| Under 50 | Imputation 1 | Imputation 2 | Imputation 3 | Imputation 4 | Imputation 5 | Imputation 6 |
| --- | --- | --- | --- | --- | --- | --- |
| Observed | 38.8% | 38.8% | 38.8% | 38.8% | 38.8% | 38.8% |
| Imputed | 10.7% | 7.1% | 7.1% | 7.1% | 10.7% | 10.7% |
| Completed | 38.4% | 38.3% | 38.3% | 38.3% | 38.4% | 38.4% |
| CFS 1-3 | Imputation 1 | Imputation 2 | Imputation 3 | Imputation 4 | Imputation 5 | Imputation 6 |
| Observed | 38.4% | 38.4% | 38.4% | 38.4% | 38.4% | 38.4% |
| Imputed | 64.3% | 75% | 75% | 75% | 67.9% | 64.3% |
| Completed | 38.8% | 39% | 39% | 39% | 38.9% | 38.8% |
| CFS 3-6 | Imputation 1 | Imputation 2 | Imputation 3 | Imputation 4 | Imputation 5 | Imputation 6 |
| Observed | 18.4% | 18.4% | 18.4% | 18.4% | 18.4% | 18.4% |
| Imputed | 17.9% | 14.3% | 14.3% | 17.9% | 17.9% | 17.9% |
| Completed | 18.4% | 18.4% | 18.4% | 18.4% | 18.4% | 18.4% |
| CFS 7-9 | Imputation 1 | Imputation 2 | Imputation 3 | Imputation 4 | Imputation 5 | Imputation 6 |
| Observed | 4.3% | 4.3% | 4.3% | 4.3% | 4.3% | 4.3% |
| Imputed | 7.1% | 3.6% | 3.6% | 0% | 3.6% | 7.1% |
| Completed | 4.4% | 4.3% | 4.3% | 4.2% | 4.3% | 4.4% |

Supplementary Material 4: Multivariable Models selected in complete case analysis

| **Candidate Factor** | **Category** | **Multivariable effect on risk of deterioration: Odds Ratio (95% CI)** | **Multivariable effect on risk of deterioration: Odds Ratio (95% CI)** | |
| --- | --- | --- | --- | --- |
| Age | Year (1 unit increase) | * | (Age/10)^3^  Fractional Polynomial | 0.997 (0.996 to 0.999 |
| GCS Vs 15 | GCS14  GCS13 | 1.5 (1.1 to 2.1)  2.7 (1.8 to 4.1) | 1.6 (1 to 2.5)  4.2 (2.4 to 7.2) | |
| Abnormal Neurological Examination | Abnormal | 1.4 (0.99 to 2.1) | 2.1 (1.3 to 3.5) | |
| Injury severity on CT  Vs simple skull fracture  (categories described in detail supplementary material 2) | 2) Complex Skull fractures  3)1-2 bleeds < 5mm (total)  4) No or minimal mass effect  5) Significant midline shift  6) High/mixed-density lesion  7) Cerebellar/Brain stem injury | 1.3 ( 0.4 to 4.5)  0.7 (0.2 to 2.2)  1.8 (0.6 to 5.4)  5.6 (1.8 to 17.5)  14.4 (4.4 to 46.6)  10.1 (2 to 49.8) | 1.3 (0.2 to 7.2)  0.6 (0.1 to 3.6)  2.3 (0.5 to 10.2)  11 (2.3 to 52)  47.4 (9.9 to 227.5)  10.5 (1.2 to 89.3) | |
| Subdural bleed | Yes | 1.8 (1.3 to 2.4) | * | |
| Extracranial Injury | ISS (1 unit increase) | * | 1.06 (1.03 to 1.1) | |
| Rockwood Frailty Score  Vs under 50 | CFS 1-3  CFS 4-6  CFS 7-9 | * | 1.4 (0.8 to 2.6)  0.6 (0.2 to 1.7)  0.1 ( 0.01 to 1.05) | |
| Preinjury Anti-coagulation or anti-platelets | Yes | 1.3 (1 to 1.8) | * | |
| Intoxicated | Yes | * | 0.6 (0.4 to 0.95) | |
| Number of Injuries on CT  Vs 1 | 2  3  4  5  Diffuse injury | * | 0.9 (0.5 to 1.5)  0.7 (0.4 to 1.4)  1.6 (0.8 to 3.1)  2.5 (1.2 to 5.1)  2.1 (0.2 to 18.4) | |
| Contusion Present | Yes | 1.3 (0.99 to 1.8) | * | |
| Extradural bleed | Yes | 1.7 (1 to 2.8) | * | |
| Intraparenchymal haemorrhage Present | Yes | * | 0.5 (0.2 to 0.9) | |
| Intra-ventricular bleed | Yes | 1.9 (0.9 to 3.9) | * | |

*Not Selected into model

Supplementary Material 5:

1. **ROC curve of derived model for primary composite outcome of deterioration for discharge from the ED**


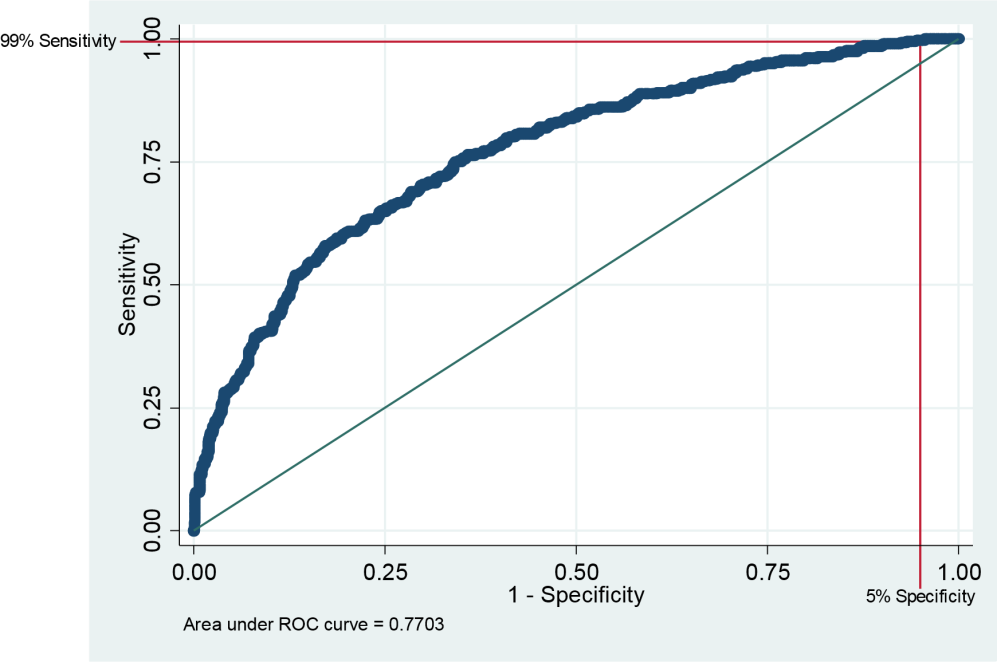


1. **ROC curve of derived model for secondary composite outcome of deterioration indicating need for specialist neurosurgical admission**


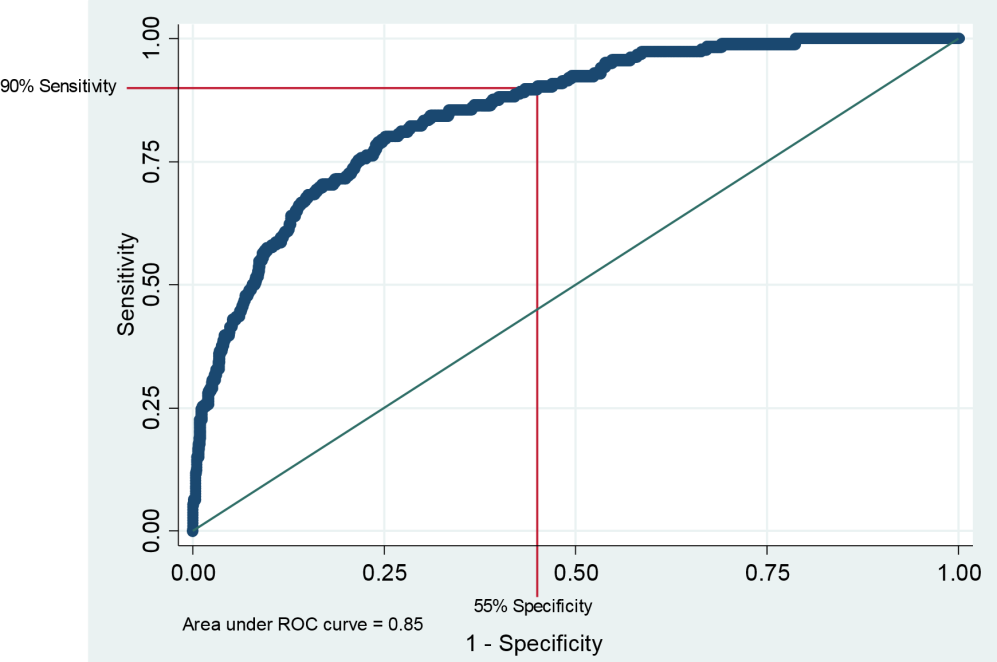


*AUC estimated in patients with complete data for explanatory variables in each model

Supplementary Material 6: Performance of risk score including Hb

| **Factor** | **Coefficient (optimism adjusted)** | **Risk Score Value** |
| --- | --- | --- |
| Preinjury Anti-coagulation or anti-platelets | 0.3 | 1 |
| GCS  15  14  13 | 0 (Vs)   \| 0.4 \| \| --- \| \| 0.7 \| | **GCS 15** 0  **GCS 14** 1  **GCS 13** 2 |
| Normal first Neurological Examination | 0.45 | **Abnormal** 1.5 |
| Number of Injuries on CT  **1**  **2**  **3**  **4**  **5**  **Diffuse** | 0 (Vs)   \| 0.25 \| \| --- \| \| 0.4 \| \| 0.8 \| \| 0.9 \| \| 0.3 \| | **1** 0  **2** 1  **3** 1  **4** 3  **5** 3  **Diffuse** 1 |
| Injury severity on CT*  **1** simple skull fracture  **2** complex Skull Fracture  **3** 1-2 bleeds < 5mm  **4** Marshall II  **5** Marshall II/IV  **6** Marshall VI  **7** Brain stem/Cerebellar | 0 (Vs)   \| 0.3 \| \| --- \| \| 0.08 \| \| 0.7 \| \| 1.7 \| \| 2.7 \| \| 1.7 \| | **1** 0  **2** 1  **3** 0  **4** 2  **5** 5  **6** 9  **7** 5 |
| ISS (body regions excluding head) | 0.2 | **Up to 2 non-significant extra-cranial injuries**** 0  **Any significant extra-cranial injury or 3 or more injuries** 2 |
| Hb | -0.01 | **Hb<10** 2 |
| Constant | -1.38 |  |

| N=1370 | **Deteriorated** | **Didn’t deteriorate** | **Positive Predictive Value (PPV)**  **Negative Predictive Value (NPV)** |
| --- | --- | --- | --- |
| **Performance of Risk score** | | | |
| Admission (Score>0) | 396 | 912 | PPV=30.3% |
| Discharge (Score=<0) | 2 | 60 | NPV=96.8% |
|  | Sensitivity = 99.5%  (95% CI: 98% to 99.9%) | Specificity= 6.2%  (95% CI: 4.8% to 7.9%) |  |

Supplementary material 7: risk stratification by risk score

| **Risk Score** | **0** | **1-5** | **>5** |
| --- | --- | --- | --- |
| Deteriorated | 2 | 181 | 242 |
| Did not deteriorate | 85 | 855 | 204 |
| Prevalence deterioration | 2.3% | 15.5% | 54% |
